# Spadin Modulates Astrocytic Passive Conductance via Inhibition of TWIK-1/TREK-1 Heterodimeric Channels

**DOI:** 10.3390/ijms21249639

**Published:** 2020-12-17

**Authors:** Yeonju Bae, Jae Hyouk Choi, Kanghyun Ryoo, Ajung Kim, Osung Kwon, Hyun-Gug Jung, Eun Mi Hwang, Jae-Yong Park

**Affiliations:** 1 School of Biosystems and Biomedical Sciences, College of Health Sciences, Korea University, Seoul 02841, Korea; dwuncle@korea.ac.kr (Y.B.); kanryoo@korea.ac.kr (K.R.); osung@korea.ac.kr (O.K.); wjdjd77@kist.re.kr (H.-G.J.); 2 Center for Functional Connectomics, Korea Institute of Science and Technology (KIST), Seoul 02792, Korea; h14006@kist.re.kr (J.H.C.); kitkim819@kist.re.kr (A.K.)

**Keywords:** astrocyte, astrocytic passive conductance, TWIK-1/TREK-1-heterodimeric channel, spadin

## Abstract

Astrocytes, the most abundant cell type in the brain, are non-excitable cells and play critical roles in brain function. Mature astrocytes typically exhibit a linear current–voltage relationship termed passive conductance, which is believed to enable astrocytes to maintain potassium homeostasis in the brain. We previously demonstrated that TWIK-1/TREK-1 heterodimeric channels mainly contribute to astrocytic passive conductance. However, the molecular identity of astrocytic passive conductance is still controversial and needs to be elucidated. Here, we report that spadin, an inhibitor of TREK-1, can dramatically reduce astrocytic passive conductance in brain slices. A series of gene silencing experiments demonstrated that spadin-sensitive currents are mediated by TWIK-1/TREK-1 heterodimeric channels in cultured astrocytes and hippocampal astrocytes from brain slices. Our study clearly showed that TWIK-1/TREK-1-heterodimeric channels can act as the main molecular machinery of astrocytic passive conductance, and suggested that spadin can be used as a specific inhibitor to control astrocytic passive conductance.

## 1. Introduction

Astrocytes are the most abundant cells in the brain, and play a wide spectrum of roles [1,2]. For a long time, astrocytes had been thought to be just assistants for neurons to perform brain functions [1,3]. However, many recent studies suggest that astrocytes and neurons dynamically share mutual interactions to modulate each other [4]. Unlike neurons expressing excitable action potentials, mature hippocampal astrocytes are electrophysiologically unexcitable and show a linear current–voltage (*I*–*V*) relationship in potassium (K^+^) conductance, termed passive conductance [5,6,7]. The passive conductance of astrocytes may contribute to the control of extracellular K^+^ concentrations, which is important for determining the survival and excitability of neurons under physiological and pathophysiological circumstances [6,8,9]. However, the molecular identities and physiological functions of astrocytic passive conductance are not fully understood.

Because of the typical linear *I–V* relationship of astrocytic passive conductance, it has been speculated that astrocytic passive conductance is carried by non-voltage-gated K^+^ channels [10]. Recently, we demonstrated that two-pore domain K^+^ channels (K2P), TWIK-1 (K2P 1.1, KCNK1) and TREK-1 (K2P 2.1, KCNK2), form a heterodimeric channel in a disulfide bond-dependent manner, and that knockdown (KD) of *TWIK-1* or *TREK-1* genes induces a huge reduction in astrocytic passive conductance [11]. In addition, quinine, a nonspecific K^+^ channel inhibitor, effectively inhibits astrocytic passive conductance by 58% at a concentration of 200 μM and potently decreases cloned TREK-1 and TWIK-1 (K274E) currents at a concentration of 100 μM [10]. These studies strongly suggest that TREK-1 and TWIK-1 comprise the major molecular machinery of passive conductance in hippocampal astrocytes. However, a more recent study reported that *TREK-1* single or *TWIK-1/TREK-1* double gene knockout (KO) mice do not exhibit altered electrophysiological properties of astrocytes [12]. Therefore, the molecular identity of astrocytic passive conductance remains to be verified.

It is well known that TREK-1 activity is modulated by multiple signals such as mechanostretching, heat, pH changes, polyunsaturated fatty acids, and Gq or Gs-induced phosphorylation [13,14,15,16,17,18,19]. TREK-1 channel activity is affected by high-dose quinine or quinidine, but not by conventional K^+^ channel blockers such as Ba^2+^, 4-aminopyrimidine (4-AP), and tetraethylammonium (TEA) [20,21,22]. Fluoxetine and paroxetine, which are clinically prescribed as antidepressants, are also effective in the reduction of TREK-1 activity [23,24,25,26]. Recently, spadin, a peptide derived from sortilin, was discovered as a highly specific blocker for TREK-1 [27]. Treatment of 100 nM spadin inhibited about 60% of the TREK-1 current stimulated by arachidonic acid in TREK-1-transfected COS-7 cells. A dose-response experiment of spadin on TREK-1 indicated an IC50 value of 70.7 nM. In addition, spadin alleviates depression phenotypes in animal behavioral models [27,28]. Although TREK-1 is highly expressed in neurons and astrocytes in many brain regions, including the hippocampus [20,29,30], most studies concerning TREK-1 inhibitors have been performed in neurons. Not surprisingly, the effects of spadin on the electrophysiological properties of astrocytes have not yet been examined. In addition, since astrocytic passive conductance is not altered in *TREK-1* KO mice, it is still unclear what the physiological function of TREK-1 in astrocytes might be due to the high-level expression of TREK-1 in astrocytes [12].

In the present study, we used spadin to examine the role of TREK-1 in terms of the electrophysiological properties of astrocytes. As a result, we found that spadin inhibited linear-like K^+^ currents in cultured astrocytes, and astrocytic passive conductance in hippocampal brain slices. Using gene silencing techniques against the TWIK-1/TREK-1 heterodimeric channel, we provide strong evidence to show that TWIK-1/TREK-1 heterodimeric channels are a main component of astrocytic passive conductance, which is specifically inhibited by spadin, a potent antidepressant.

## 2. Results

### 2.1. Spadin Inhibits TREK-1 Mediated K^+^ Currents in Cultured Astrocytes

**Figure 1:**
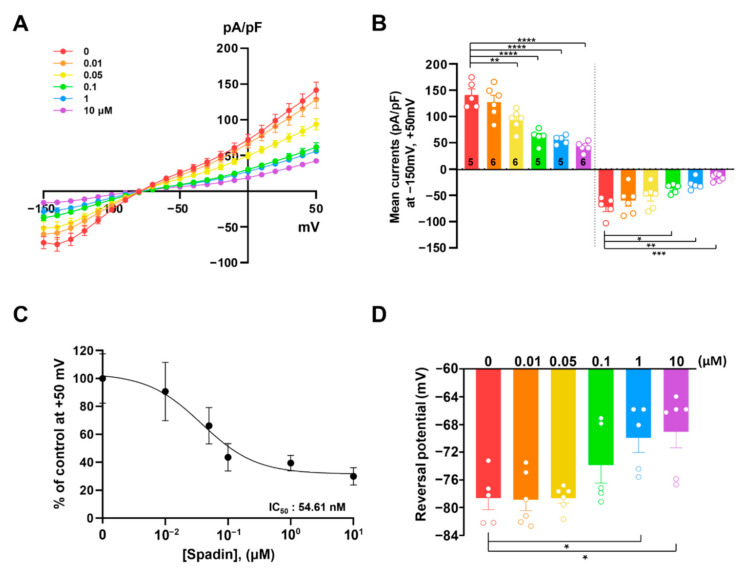
Spadin inhibits K^+^ currents in cultured astrocytes in a concentration-dependent manner. (**A**) Current densities induced by voltage ramping were recorded in cultured astrocytes before and after application of spadin. Whole-cell recording showed that K^+^ currents were inhibited by spadin in a concentration-dependent manner. (**B**) Bar graph showing K^+^ currents averaged from results in (**A**) at +50 mV and −150 mV. The number on each bar indicates *n* for each condition. (**C**) Spadin concentration–response curves to outward currents at +50 mV. (**D**) Reversal potential before and after spadin treatment. Data are obtained from results in (**A**). All values are mean ± standard error of the mean (s.e.m). *p*-values were obtained with one-way ANOVA followed by Turkey’s post hoc test. * *p* < 0.05, ** *p* < 0.01, *** *p* < 0.001, and **** *p* < 0.0001.

TREK-1 is highly expressed in astrocytes [20,29,30]. In order to examine the contribution of TREK-1 to the electrophysiological properties of astrocytes with pharmacological tools, we first examined the effects of spadin, a specific inhibitor of TREK-1, on the background conductance in cultured astrocytes. Consistent with a previous report [11], cultured astrocytes displayed an *I–V* relationship with a linear-like shape, consisting of both outwardly rectifying outward current and weakly rectifying inward current (Figure 1A). When astrocytes were treated with spadin over a series of concentrations, both the outward and inward currents of astrocytes were gradually reduced as spadin concentration increased (Figure 1A,B). As shown in Figure 1C, the IC_50_ value of spadin on astrocytic conductance was 54.65 nM. In addition, spadin treatment showed a positive shift from –78.4 ± 1.7 mV to −69.9 ± 2.3 mV in reversal membrane potential (RMP) of cultured astrocytes in a concentration dependent manner, which suggested that the membrane potential of astrocytes is depolarized by spadin treatment (Figure 1D). Although spadin is reported as a specific inhibitor of the TREK-1 channel [27], we could not completely exclude the possibility of the involvement of other unknown channels. Nevertheless, these data strongly suggested that spadin dramatically reduced linear-like conductance in cultured astrocytes.

**Figure 2:**
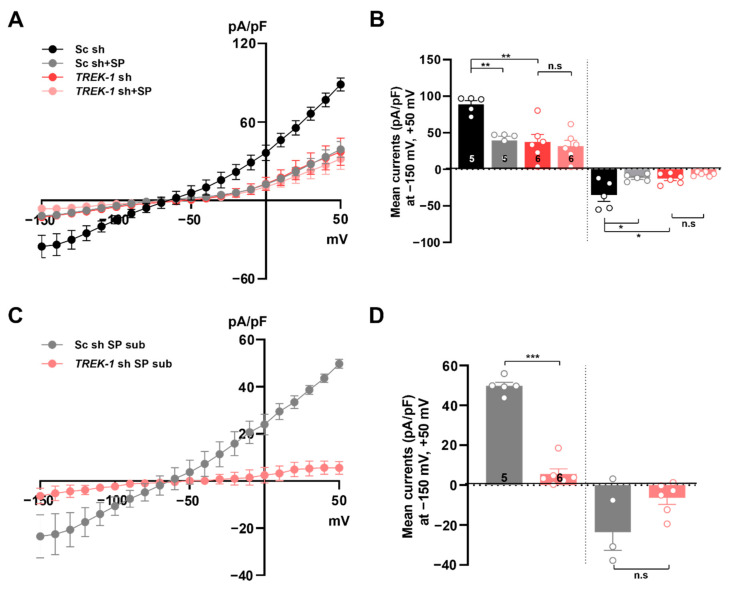
Spadin inhibits TREK-1 mediated current in cultured astrocytes. (**A**) Current densities induced by voltage ramping were measured before and after application of 10 µM spadin (SP) in astrocytes transfected with scrambled control (Sc) short hairpin-forming interfering RNA (shRNA) (Sc sh) or *TREK-1* shRNA. (**B**) Summary bar graph showing averaged currents with or without spadin treatment at +50 mV and −150 mV in (**A**). The number on each bar indicates *n* for each condition. All values are mean ± s.e.m. *p*-values were obtained with one-way ANOVA followed by Turkey’s post hoc test. n.s: not significant, * *p* < 0.05 and ** *p* < 0.01. (**C**) Spadin-sensitive currents (SP sub) were calculated by the difference between currents before and after spadin application in astrocytes in (**A**). (**D**) Bar graph indicates spadin-sensitive currents averaged from results in (**C**) at +50 mV and −150 mV. The number on each bar indicates *n* for each condition. All values are mean ± s.e.m. *p*-values were obtained with Student’s *t*-test. n.s: not significant, *** *p* < 0.001.

Since both the *I–V* relationship and RMP are mainly dependent on K^+^ ions, and spadin is a specific TREK-1 inhibitor [27], it seems that spadin-sensitive linear-like conductance involves TREK-1-mediated K^+^ conductance in cultured astrocytes. To confirm the inhibitory effects of spadin on TREK-1-mediated K^+^ conductance in cultured astrocytes, we next examined whether gene silencing of endogenous *TREK-1* with *TREK-1*-specific short hairpin-forming interfering RNA (shRNA) could affect the inhibitory effect of spadin on astrocytic K^+^ conductance. The specificity of *TREK-1* shRNA has been documented previously [11,31]. Treatment with *TREK-1* shRNA caused dramatic reductions in both outward and inward currents of astrocytes in comparison to the scrambled control shRNA (Sc shRNA) treatment in the absence of spadin. Interestingly, spadin did not affect K^+^ conductance in astrocytes treated with *TREK-1* shRNA, but dramatically reduced currents in control cells (Figure 2A,B). We measured spadin-sensitive K^+^ currents in astrocytes by subtracting K^+^ currents from before treatment of spadin with those from after treatment with spadin. Sc shRNA-treated control astrocytes exhibited relatively large spadin-sensitive K^+^ current densities (88.74 ± 5.04 at +50 mV), with a linear-like topology of the *I*–*V* relationship. In contrast, spadin-sensitive K^+^ conductance in astrocytes transfected with *TREK-1* shRNA was almost negligible (Figure 2C,D). These results strongly suggested that spadin-sensitive K^+^ currents showed a linear-like *I*–*V* relationship, which was mediated by TREK-1 channels in cultured astrocytes.

### 2.2. Spadin Inhibits TWIK-1 Mediated K^+^ Currents in Cultured Astrocytes

We previously showed that TREK-1 can form heterodimeric channels with TWIK-1 by inter-molecular disulfide bonds, and the TWIK-1/TREK-1 heterodimeric channel plays a critical role in mediating astrocytic passive conductance [11]. Therefore, we next examined whether TWIK-1 dependent K^+^ conductance might also be affected by spadin treatment of astrocytes. The specificity of *TWIK-1* shRNA has been previously documented [11]. As expected, when we silenced TWIK-1 expression by *TWIK-1* shRNA, K^+^ conductance was prominently diminished in cultured astrocytes, and spadin did not affect K^+^ conductance in astrocytes treated with *TWIK-1* shRNA. However, K^+^ conductance was drastically reduced by spadin treatment of astrocytes transfected with Sc shRNA (Figure S1A,B). We also found that spadin-sensitive K^+^ conductance almost disappeared in the presence of *TWIK-1* shRNA in cultured astrocytes (Figure S1C,D).

Furthermore, when both *TREK-1* and *TWIK-1* shRNAs were used for double KD of *TREK-1* and *TWIK-1* in primary cultured astrocytes, K^+^ conductance was reduced to similar levels to those under each single KD of the channels, and spadin did not cause any further current reduction (Figure S1E,F). Furthermore, spadin-sensitive K^+^ conductance mostly disappeared in astrocytes showing double KD of *TREK-1* and *TWIK-1* (Figure S1G,H). These results clearly showed that spadin-sensitive K^+^ conductance is transduced by TWIK-1/TREK-1 heterodimeric channels in cultured astrocytes.

### 2.3. Knockout of TREK-1 or TWIK-1 with CRISPR/Cas9 System Abolishes Spadin-Sensitive Currents in Cultured Astrocytes

**Figure 3:**
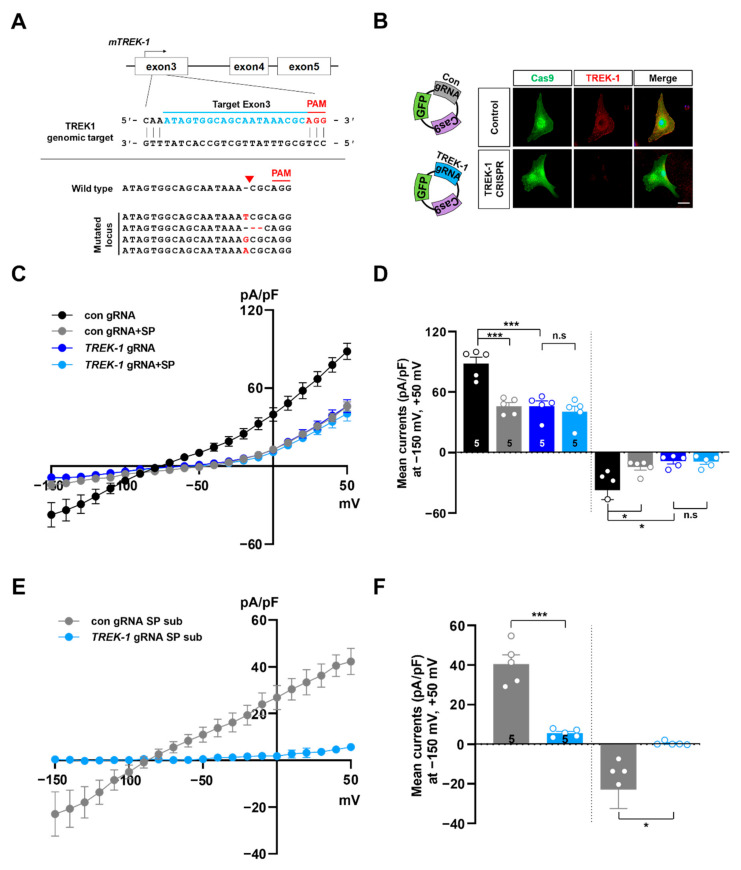
Spadin’s effects on astrocytic K^+^ current were abrogated by genetic ablation of *TREK-1* using clustered regularly interspaced short palindromic repeats (CRISPR)/Cas9. (**A**) Schematic representation of the genomic structure of the *TREK-1* gene. Target sequences of gRNA are located within exon3 of mouse *TREK-1* gene. Examples of indel mutations are displayed. (**B**) Immunocytochemical images of control gRNA (Con gRNA) or *TREK-1* gRNA transfected cultured astrocytes stained with anti-TREK-1 antibody. (**C**) Current densities showing before and after application of 10 µM spadin (SP) to control gRNA or *TREK-1* gRNA transfected astrocytes. (**D**) Summary bar graph of control gRNA or *TREK-1* gRNA currents with or without spadin (SP) plotted at +50 mV and −150 mV. The number on each bar indicates *n* for each condition. All values are mean ± s.e.m. *p*-values were obtained with one-way ANOVA followed by Turkey’s post hoc test. n.s: not significant, * *p* < 0.05 and *** *p* < 0.001. (**E**) Subtract spadin (SP-sub) treatment currents from before application of spadin in control gRNA or *TREK-1* gRNA transfected astrocytes. (**F**) Summary bar graph of control gRNA (Con gRNA-SP sub) or *TREK-1* gRNA (*TREK-1* gRNA-SP sub) spadin-subtracted currents plotted at +50 mV and −150 mV. The number on each bar indicates *n* for each condition. All values are mean ± s.e.m. *p*-values were obtained with Student’s *t*-test. * *p* < 0.05 and *** *p* < 0.001.

Since gene KD with shRNA could induce off-target effects [32], we confirmed that the spadin-sensitive currents of astrocytes were mediated by the TWIK-1/TREK-1 heterodimeric channel with an additional genome editing tool, the clustered regularly interspaced short palindromic repeats (CRISPR)/CRISP-associated endonuclease 9 (Cas9) [33,34]. We designed a single guide RNA (sgRNA) targeting exon 3 of the *TREK-1* gene and co-expressed *TREK-1*-targeting sgRNA and Cas9 (spCas9; Cas9 from Streptococcus pyogenes) gene in cultured astrocytes to inactivate *TREK-1* at the cellular level. The CRISPR/Cas9 system is a powerful tool to edit genes in replicating eukaryotic cells, resulting in frame-shifting insertion/deletion (indel) mutations and subsequent protein depletion [35]. To assess *TREK-1* modification in targeted astrocytes, we sequenced the *TREK-1* locus using PCR-based genomic DNA sequencing analysis at the single cell level. We found that about 88% of targeted astrocytes (39 cells among 44 cells) contained indel mutations two days after transfection of the CRISPR/Cas9 vector (Figure 3A). Expression of TREK-1 protein was drastically reduced, so it was difficult to detect channels by immunocytochemistry in *TREK-1* KO astrocytes (Figure 3B). Through electrophysiological recording, we found that *TREK-1* KO astrocytes had decreased K^+^ conductance as compared to control astrocytes. Spadin did not engender further differences in K^+^ conductance in KO astrocytes, but did result in a huge reduction in control cells (Figure 3C,D). Control cells showed a linear relationship between membrane potential and spadin-sensitive K^+^ conductance, but KO cells lost almost all spadin-sensitive K^+^ conductance (Figure 3E,F). These results from both *TREK-1* KD and KO experiments suggested that TREK-1-mediated K^+^ currents mainly contribute to the linear-like topology of background K^+^ conductance, and are specifically inhibited by spadin in astrocytes.

**Figure 4:**
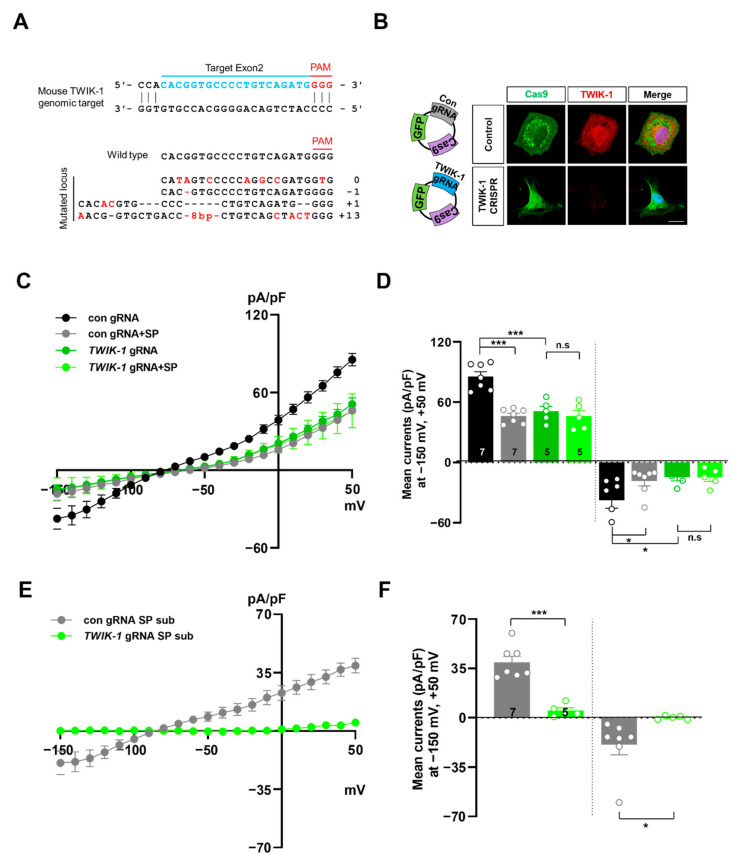
Spadin inhibits TWIK-1 mediated K^+^ currents in a TREK-1-dependent manner in cultured astrocytes. (**A**) Schematic representation of the genomic structure of the *TWIK-1* gene. Target sequences of gRNA are located within exon2 of mouse *TWIK-1* gene. Examples of indel mutations are displayed. (**B**) Immunocytochemical images of control gRNA (Con gRNA) or *TWIK-1* gRNA transfected cultured astrocytes stained with anti-TWIK-1 antibody. (**C**) Current densities before and after application of 10 µM spadin (SP) to control gRNA or *TWIK-1* gRNA transfected astrocytes. (**D**) Summary bar graph of control gRNA or *TWIK-1* gRNA currents with or without spadin (SP) plotted at +50 mV and −150 mV. The number on each bar indicates *n* for each condition. All values are mean ± s.e.m. *p*-values were obtained with one-way ANOVA followed by Turkey’s post hoc test. n.s: not significant, * *p* < 0.05 and *** *p* < 0.001. (**E**) Subtract spadin (SP-sub) treatment currents from before application of spadin currents in control gRNA or *TWIK-1* gRNA transfected astrocytes. (**F**) Summary bar graph of con gRNA (Con gRNA-SP sub) or *TWIK-1* gRNA (*TWIK-1* gRNA-SP sub) spadin-subtracted currents plotted at +50 mV and −150 mV. Th number on each bar indicates *n* for each condition. All values are mean ± s.e.m. *p*-values were obtained with Student’s *t*-test. * *p* < 0.05 and *** *p* < 0.001.

Next, we knocked-out *TWIK-1* gene expression using CRISPR/Cas9 gene silencing techniques, and confirmed the KO efficiency for *TWIK-1* by immunostaining with an antibody against TWIK-1 protein (Figure 4A,B). After genetic ablation of *TWIK-1*, K^+^ conductance was dramatically decreased compared with that of control astrocytes, and spadin could not induce any further changes to K^+^ conductance. In control astrocytes, spadin induced a wide range of decreases in K^+^ conductance (Figure 4C,D). Spadin-sensitive currents in *TWIK-1* KO astrocytes almost disappeared, which showed a similar tendency to that in *TREK*-*1* KO astrocytes (Figure 4E,F). These results clearly confirmed that spadin-sensitive K^+^ conductance is transduced by TWIK-1/TREK-1 heterodimeric channels in cultured astrocytes.

### 2.4. Spadin Inhibits Concatenated TWIK-1-TREK-1 Heterodimers in Heterologous Expression System

**Figure 5:**
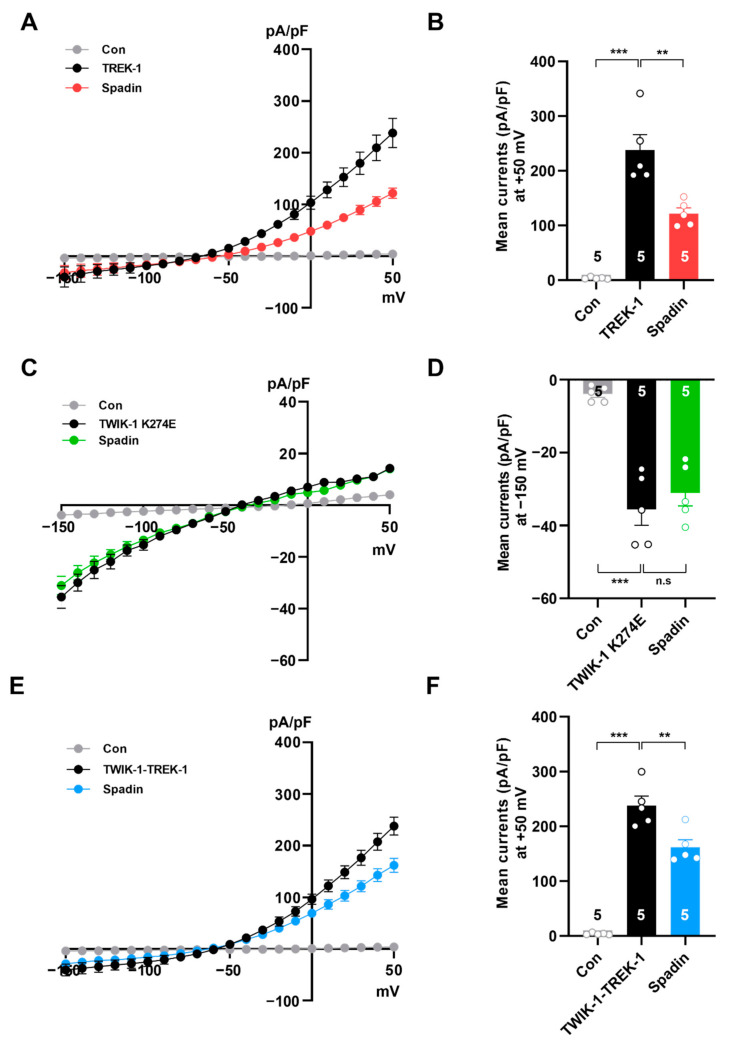
Spadin regulates K^+^ currents mediated by TWIK-1/TREK-1 heterodimeric channels. Current densities induced by voltage ramping were measured in COS-7 cells transfected with control vector (Con) or several channel expressing vectors. (**A**,**C**,**E**) Current densities induced by voltage ramping were measured in COS-7 cells transfected with TREK-1 (**A**), TWIK-1 (K274E) (**C**), and TWIK-1/TREK-1 (**E**) before and after treatment with 10 µM spadin, respectively. Summary histograms show averaged current densities recorded from cells expressing TREK-1 (**B**) and TWIK-1/TREK-1 (**F**) with or without spadin treatment at +50 mV. The number on each bar indicates *n* for each condition. (**D**) TWIK-1(K274E)-mediated current densities were averaged from the plots in (**C**) at −150 mV. The number on each bar indicates *n* for each condition. All values are mean ± s.e.m. *p*-values were obtained with one-way ANOVA followed by Turkey’s post hoc test. n.s: not significant, ** *p* < 0.01 and *** *p* < 0.001.

It has been reported that spadin is highly specific for the TREK-1 channel, and that other TREK family members, such as TREK-2 and TRAAK homodimer channels, are not sensitive to spadin [36,37]. Interestingly, a recent study showed that spadin can also inhibit TREK-1/TREK-2 heterodimeric channels [36]. Therefore, we proposed two possibilities to explain how the TWIK-1 channel is affected by spadin in astrocytes. First, the TWIK-1 channel may be regulated by spadin in a direct manner, similar to the TREK-1 channel. Second, even if the TWIK-1 channel is not directly affected by spadin, TWIK-1/TREK-1 heterodimeric channels may be regulated in a TREK-1-dependent manner. To test the first hypothesis, we compared the effects of spadin on K^+^ currents mediated by the TREK-1 and TWIK-1 homodimer channel, respectively (Figure 5A,C). Because wild-type TWIK-1 channels are known to mediate very small currents, it is particularly difficult to detect TWIK-1-mediated currents in a heterologous system. Therefore, we transfected COS-7 cells with TREK-1 or TWIK-1(K274E), a mutant of TWIK-1, which has greater K^+^ currents than the wild-type TWIK-1 channel [38]. Next, we examined the effects of spadin on K^+^ conductance. K^+^ conductance was very low in control vector-transfected COS-7 cells (Figure 5A,C,E). TREK-1-mediated K^+^ conductance was markedly inhibited, by about 50% at +50 mV, by spadin treatment, whereas TWIK-1(K274E)-mediated currents were not affected (Figure 5A–D). Next, to test the second possibility, we generated a DNA construct encoding a concatenated TREK-1-TWIK-1 heterodimeric channel [11]. K^+^ conductance mediated by the concatenated heterodimeric channel was largely inhibited by spadin treatment (Figure 5E,F). Based on these data, we concluded that TWIK-1 channels are not directly affected by spadin as a homodimer, but inhibited as heterodimeric channels with TREK-1. These results also strongly suggested that spadin can regulate TWIK-1/TREK-1 heterodimeric channel-mediated K^+^ currents by inhibition the TREK-1 subunit in astrocytes.

### 2.5. Spadin Inhibits Astrocytic Passive Conductance in Hippocampal Slices

**Figure 6:**
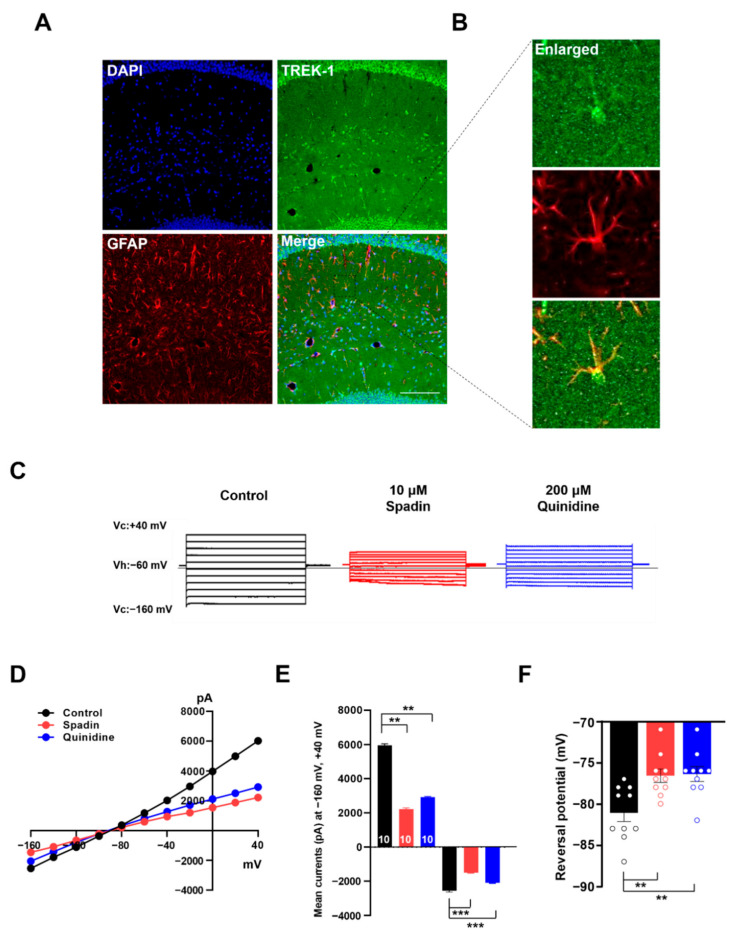
Spadin efficiently controls astrocytic passive conductance in hippocampus. (**A**,**B**) Representative fluorescence images of dual immunostaining of TREK-1 (green) and GFAP (red), with DAPI staining of astrocyte nuclei (blue) in mouse hippocampus. The images show that TREK-1 was highly expressed in astrocytes. Scale bar, 150 μm. (**C**) Representative traces of passive conductance induced by voltage stepping from −160 mV to +40 mV in hippocampal astrocytes treated with vehicle (black), 10 μM spadin (red) or 200 μM quinidine (blue) respectively. (**D**) Current–voltage (*I*–*V)* curves of passive conductance in (**C**). (**E**,**F**) Bar graphs showing averaged currents at −160 mV and +40 mV of holding potentials (**E**) and reversal potentials (Vrev) (**F**), respectively. The number on each bar indicates *n* for each condition. All values are mean ± s.e.m. *p*-values were obtained with one-way ANOVA followed by Turkey’s post hoc test. ** *p* < 0.01 and *** *p* < 0.001.

Passive conductance is one of the most important electrophysiological characteristics of mature hippocampal astrocytes [39,40,41]. However, selective inhibitors of astrocytic passive conductance have not yet been reported, except for high concentrations of quinine [10]. Therefore, based on our present results, we aimed to measure the effects of spadin on astrocytic passive conductance in mouse hippocampal slices. Immunohistochemical staining clearly showed high expression of TREK-1 in astrocytes from mouse hippocampal slices (Figure 6A). Electrophysiological recording clearly showed that spadin treatment (10 μM) induced a very substantial reduction in astrocytic passive conductance (Figure 6C). Both the outward current at +40 mV and inward current at −160 mV diminished to 30% and 40% of the control, respectively (Figure 6E). The *I*–*V* relationship maintained linearity, and the slope became much gentler with spadin application (Figure 6D). Reversal potential was slightly depolarized from −81.1 ± 1.05 mV to −76.6 ± 0.82 mV with spadin treatment (Figure 6F). Quinidine, another inhibitor of TREK-1, also induced a similar reduction in passive conductance and reversal potential in astrocytes (Figure 6C–F). Interestingly, quinidine was required at a much higher concentration than spadin to obtain a similar inhibitory effect on astrocytic passive conductance. These results suggested that spadin can be used as an efficient inhibitor of astrocytic passive conductance.

### 2.6. Spadin Specifically Inhibits TWIK-1/TREK-1 Heterodimeric Channel-Mediated Astrocytic Passive Conductance

**Figure 7:**
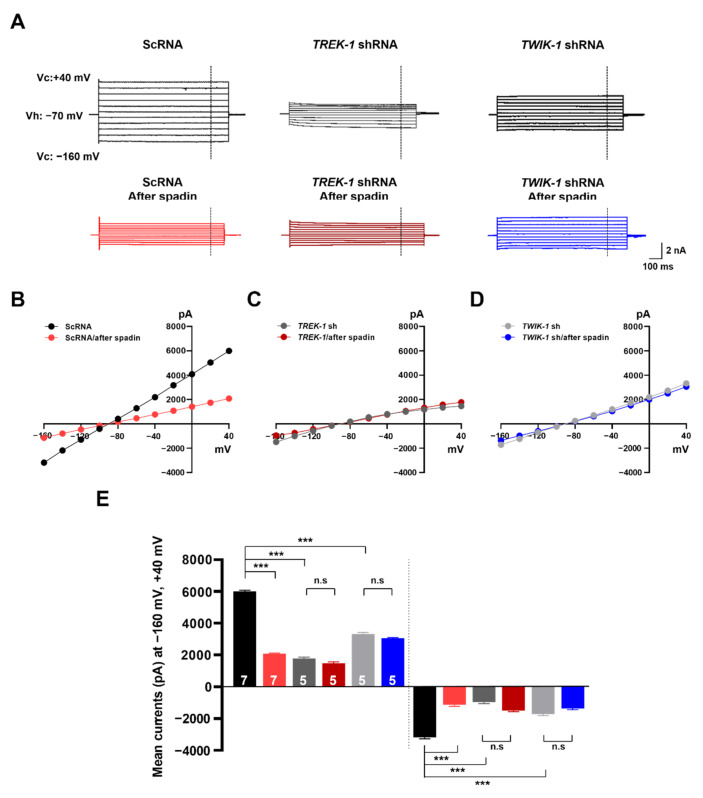
Spadin does not affect astrocytic passive conductance in mice with *TREK-1* or *TWIK-1* knockdown (KD). (**A**) Representative traces of passive conductance induced by voltage stepping from −160 mV to +40 mV in hippocampal astrocytes after injection with lentiviruses expressing scrambled control shRNA (ScRNA), *TWIK-1* shRNA, or *TREK-1* shRNA in the absence or presence of 10 µM spadin, respectively. (**B**–**D**) I–V curves of passive conductance in astrocytes infected with ScRNA (**B**), *TWIK-1* shRNA (**C**), or *TREK-1* shRNA (**D**), respectively. The curves obtained from hippocampi treated with spadin are presented in different colors (red, blue, or purple). (**E**) The bar graph shows averaged current at −160 mV and +40 mV of holding potentials. Th number on each bar indicates *n* for each condition. All values are mean ± s.e.m. *p*-values were obtained with one-way ANOVA followed by Turkey’s post hoc test. n.s: not significant and *** *p* < 0.001.

Furthermore, we tested whether the inhibitory effect of spadin on astrocytic passive conductance is dependent on TWIK-1/TREK-1 heterodimeric channels. To elucidate this, we made lentivirus carrying shRNAs targeting *TWIK-1* or *TREK-1* (Lenti-*TWIK-1* shRNA or Lenti-*TREK-1* shRNA) and scrambled control RNA (Lenti-ScRNA), respectively [11]. After two weeks of stereotaxic injection of the lentiviruses into mouse hippocampus, we measured astrocytic passive conductance in hippocampal slices with or without spadin treatment using electrophysiological recording. In hippocampal astrocytes infected with Lenti-ScRNA, passive conductance was drastically decreased by spadin treatment. In contrast, astrocytes infected with Lenti-*TWIK-1* shRNA or Lenti-*TREK-1* shRNA showed largely diminished passive conductance, even without spadin, and this reduced conductance was not further affected by spadin application (Figure 7A). Spadin diminished the slope of the *I*–*V* relationship without any differentiation in the linearity (Figure 7B). This suggested that spadin inhibits the activity of channels that mediate astrocytic passive conductance. Gene silencing of *TREK-1* or *TWIK-1* reduced the slope of *I*–*V* curves, and spadin did not provoke any further changes (Figure 7C–E). This finding provides strong evidence that spadin-sensitive astrocytic passive conductance is mediated by TWIK-1/TREK-1 heterodimeric channels, which represent the major contributor to astrocytic passive conductance.

## 3. Discussion

Astrocytes, non-excitable cells, express many passages for ion transduction, such as ion transporters and ion channels [4]. In electrophysiology, astrocytes are hyperpolarized in the resting state, and pass large currents specific to K^+^ ions across the cytoplasmic membrane, termed passive conductance [5,6,7]. In this study, we found that spadin, a specific inhibitor of TREK-1, efficiently reduced TWIK-1/TREK-1 heterodimeric channel-mediated K^+^ currents in cultured astrocytes, and controlled astrocytic passive conductance in hippocampal brain slices.

We previously demonstrated that TWIK-1/TREK-1 heterodimeric channels mediate astrocytic passive conductance, which was confirmed by the KD of *TWIK-1* or *TREK-1* channel with their specific shRNAs [11,31]. Our present study also provides strong evidence that TWIK-1/TREK-1 heterodimeric channels mainly contribute to K^+^ currents in cultured astrocytes, and to astrocytic passive conductance, which was clearly confirmed by pharmacological silencing with spadin (Figure 1 and Figure 6) and genetic ablation of *TREK-1* or *TWIK-1* by KD or CRISPR/Cas9-mediated KO (Figure 2, Figure 3, Figure 4 and Figure 7). Our study also demonstrated that spadin can be used as an efficient and specific inhibitor of astrocytic passive conductance (Figure 6), compared to quinidine, a previously reported efficient inhibitor of astrocytic passive conductance [10].

In this study, we observed a residual conductance of some 30% that was unaccounted for by 10 µM spadin, which suggested that other spadin-insensitive K^+^ channels may contribute (Figure 6 and Figure 7). Interestingly, a recent study reported that *TREK-1* KO mice show no alteration in terms of the electrophysiological properties of astrocytes [12], while the inhibitory effects of spadin on TREK-1 were confirmed in hippocampal pyramidal neurons in *TREK-1* KO mice [27]. Based on our results and other reports [11,27] regarding the inhibitory effects of spadin on TREK-1 homodimeric and TREK-1-containing heterodimeric channels [36,37], we speculated that astrocytic passive conductance in *TREK-1* KO mice can be functionally compensated by unknown mechanisms. In general, genetic compensation in response to gene KO is a widespread phenomenon, and there are many mouse models that show phenotypic differences between KOs and KDs [42]. Therefore, other spadin-insensitive K^+^ channels might be involved in spadin-insensitive conductance, or the compensation of astrocytic passive conductance in *TREK-1* KO mice. Further studies will be needed to elucidate other spadin-insensitive K^+^ channels, which will be helpful to fully understand astrocytic passive conductance.

In several previous lines of studies, spadin was shown to reduce the current mediated by TREK-1 in heterologous systems [27,43,44]. Both human and mouse TREK-1 channels are inhibited by spadin at low concentrations [27,43,44]. However, these studies show that the effect of spadin on TREK-1 activity is enhanced by arachidonic acid (AA), but spadin effect on basal activity of such channels is unknown. In this study, we found that spadin inhibits leak K^+^ currents in cultured astrocytes without any stimulation (Figure 1) and also diminishes astrocytic passive conductance in hippocampal slices in the resting state (Figure 6). This observation suggests that spadin may play a role as a regulator for basal activity of TWIK-1/TREK-1 heterodimeric channels in astrocytes. Although the detailed electrical properties of TWIK-1/TREK-1 heterodimeric channels are still obscure, it is well known that TREK-1 channels are activated by various stimuli including AA, volatile anesthetics, acidic pH, heat, and physical stretching forces [13,14,15,16]. Thus, the activity of TWIK-1/TREK-1 heterodimeric channels can also be regulated by diverse stimuli. From this notion, it seems that spadin can regulate astrocytic passive conductance by modulating the activity of TWIK-1/TREK-1 heterodimeric channels under normal physiological conditions, as well as elevated activation under unusual conditions, such as pathological circumstances.

Spadin dramatically inhibits the TREK-1 channel, but does not affect other isoforms of K2P channels, such as TREK-2, TRAAK, TRESK, and TASK-1 in heterologous systems [28]. Meanwhile, spadin reduces acid-induced activity of TREK-1/TREK-2 tandem heterodimeric channels [36]. The activity of TWIK-1 homodimeric channels was not modulated by spadin, but concatenated TWIK-1-TREK-1 heterodimeric channels were inhibited (Figure 5). Since K2P heterodimeric channels are known to be regulated by regulators specific only for single subunit isoforms [11,36,45,46,47,48,49], even if the TWIK-1 isoform is not modulated, astrocytic passive conductance mediated by TWIK-1/TREK-1 heterodimeric channels could be regulated by spadin, a specific TREK-1 inhibitor (Figure 6 and Figure 7). From these results, it is also possible that other TREK-1 inhibitors such as fluoxetine, curcumin, SID1900, and TKCD can affect TWIK-1/TREK-1 mediated astrocytic passive conductance, which should be examined in further studies [24,50,51,52].

Several previous studies have demonstrated that TREK-1 is involved in depression [23] and TREK-1 inhibitors have been considered to have antidepressant effects [23,27,50,51,52,53]. Clearly, antidepressant effects have been reported in both *TREK-1* KO mice and mice treated with spadin, a specific TREK-1 inhibitor [23,53]. Recently, we also reported specific KD of *TREK-1* in the hippocampal neurons revealed antidepressant effects [54], suggesting that inhibition of TREK-1 in neurons is important for antidepressant phenotypes. However, in the present study, we clearly demonstrated that astrocytic TREK-1 is critical for passive conductance, and spadin, a potent antidepressant drug, can efficiently inhibit astrocytic passive conductance. Therefore, it is plausible that astrocytic TREK-1 and astrocytic passive conductance may be involved in antidepressant effects observed in *TREK-1* KO mice, and in spadin-treated mice, although the antidepressant action of TREK-1 has been generally regarded as a neurocentric mechanism. To determine whether TWIK-1/TREK-1 mediated astrocytic passive conductance is involved in depression, future studies with conditional *TREK-1* KO mice will be required.

In conclusion, our results showed that spadin, a potent antidepressant drug, can act as an efficient inhibitor of astrocytic passive conductance. Furthermore, pharmacological inhibition and genetic silencing tools strongly suggest that TWIK-1/TREK-1 heterodimeric channels mainly contribute to astrocytic passive conductance. The new concepts and tools developed in our study should be useful for understanding the functional roles of astrocytic passive conductance in various physiological and pathological conditions.

## 4. Materials and Methods

### 4.1. Chemicals

Quinidine was purchased from Sigma Aldrich (Sigma Aldrich, St. Louis, MO, USA). Spadin was purchased from Tocris (Tocris Bioscience, Bristol, UK). All substances were stored as stock solutions at −20 °C and diluted to the required concentrations in standard bath solution immediately prior to experimentation.

### 4.2. Construction of Expression Vectors

cDNAs encoding for full-length mouse *TREK-1* (GenBank accession no. NM_010607) and mouse *TWIK-1* (NM_008430) were obtained by using an RT–PCR based gateway cloning (ThermoFisher, Waltham, MA, USA). *TWIK-1 K274E* mutant was generated using full-length cDNA as templates via the EZchange site-directed mutagenesis kit (Enzynomics, Daejeon, Korea). All constructs were cloned into pDEST-IRES2-GFP vectors by gateway cloning. The concatenated *TWIK-1*-*TREK-1* were re-cloned into pDONR207 P1P5R and pDONR207 P5P2 vectors respectively via two independent BP-reactions (ThermoFisher, Waltham, MA, USA), and then performing a MultiSite Gateway LR recombination reaction (ThermoFisher, Waltham, MA, USA) according to the manufacturer’s guidelines, combining pDONR207-*TREK-1*, pDONR207-*TWIK-1* and pDEST-IRES2-GFP.

### 4.3. Construction of shRNAs

For gene silencing in cultured astrocytes and mouse brain slices, *TWIK-1* shRNA and *TREK-1* shRNA were constructed and the knockdown efficiencies of these shRNAs were validated as described previously [11,31]. Briefly, the target region of shRNA was as follows: mouse *TWIK-1*: 5′-GCATCATCTACTCTGTCATCG-3′; mouse *TREK-1*: 5′-GCGTGGAGATCTACGACAAGT-3′. These target sequences were inserted into lentiviral pSicoR vector (Addgene okasnud #11579) [55] and Scrambled shRNA-containing pSicoR construct was used as a control. These pSicoR vectors were transfected into primary cultured astrocytes to perform electrophysiological experiments. Transfected primary astrocytes were easily detected under a fluorescence microscope since GFP fluorescent protein is expressed from these pSicoR vectors [55].

### 4.4. Primary Cortical Astrocytes Culture and Transfection

Primary cortical astrocytes were prepared from one day-old male and female C57BL/6 mouse pups culture astrocytes, P1 C57BL/6 J mouse pups [11]. The cerebral cortex was dissected free of adherent meninges, minced and dissociated into single-cell suspension by trituration through a pipette. All experimental procedures described were performed in accordance with the guidelines of Korea University (Seoul, Korea). Dissociated cells were plated onto either 12 mm glass coverslips or six-well plates coated with 0.1 mg/mL Poly-d-Lysine (PDL). Cells were grown in Dulbecco’s modified Eagle’s medium (DMEM, ThermoFisher, Waltham MA, USA) supplemented with 10% heat-inactivated fetal bovine serum, 10% heat-inactivated horse serum, and penicillin–streptomycin. Cultures were maintained at 37 °C in a humidified 5% CO_2_ incubator. On the third day of culture, cells were washed by repeated pipetting and the media was replaced to remove debris and other floating cells. On the next day (fourth day of culture), the cells were replaced onto glass coverslips (1 × 104 per coverslip) coated with 0.1 mg/mL PDL. During this procedure, cultured astrocytes were transfected with various shRNAs or gRNAs with an optimized voltage protocol (1100 V, 20 pulse width ms, 2 pulses number) using the Neon Electroporation instrument (ThermoFisher, Waltham, MA, USA).

### 4.5. COS-7 Cells Culture and Transfection

COS-7 cells were purchased from the Korean Cell Line Bank (Seoul National University, Seoul, Korea) and cultured in RPMI 1640 supplemented with 10% fetal bovine serum and penicillin–streptomycin at 37 °C in a humidified 5% CO_2_ incubator. One day prior to performing electrophysiological experiments, COS-7 cells were transfected with several expression vectors. Transfection experiments were performed with Lipofectamine 2000 (ThermoFisher, Waltham, MA, USA), according to the manufacturer’s protocol.

### 4.6. KO by CRISPR/Cas9 System

*TREK-1* and *TWIK-1* were knocked-out in primary cultured astrocytes using CRISPR/Cas9 technology. *TREK-1, TWIK-1*, and control CRISPR/Cas9 plasmid were purchased from Santa Cruz Biotechnology (Santa Cruz, Dallas, TX, USA; sc-421244, sc-421243, and sc-418922, respectively). Briefly, all plasmids were transfected by electroporation into primary cultured astrocytes. Transfected astrocytes were plated in 60 mm dishes and cultured for 48 h. When different alleles of mutant clones had to be analyzed, target regions were amplified by PCR using 2× TOPsimpleTM DyeMIX-Forte (Enzynomics, Daejeon, Korea), amplicons were cloned using pTOP Blunt V2 (Enzynomics, Daejeon, Korea), and transformed into TOP10 competent cells. Bacterial colonies containing plasmids with inserts were selected by colony-direct PCR, and PCR products were confirmed by DNA sequencing analysis.

### 4.7. Immunocytochemistry

Primary astrocytes were seeded onto Poly-D-Lysine (PDL)-coated coverslips and maintained in a humidified incubator for 2 days. Thereafter, astrocytes were transfected with the indicated CRISPR/Cas9 plasmids by electroporation and incubated for 48 h. Cells were fixed with 4% paraformaldehyde (PFA) and permeabilized with 0.3% Triton X-100. After blocking for 1 h in 0.1% Triton X-100 and 3% BSA, the cells were incubated with mouse anti-TREK-1 (Santa Cruz Biotechnology, Dallas, TX, USA; RRID: sc-398449, 1:100) or mouse anti-TWIK-1 (Santa Cruz Biotechnology, Dallas, TX, USA; RRID: sc-517040, 1:100) antibodies at 4 °C overnight. After washing with PBS, cells were incubated with Alexa Fluor 594-conjugated secondary antibodies (Jackson Immunoresearch, West Grove, PA, USA, 1:400) for 1 h at room temperature. After three rinses with PBS and staining with 4′,6-diamidino-2-phenylindole (DAPI) to visualize nuclei, images were acquired by confocal microscopy using a Nikon A1 confocal microscope (Nikon Instruments Inc., Melville, NY, USA).

### 4.8. Electrophysiological Recording in Cultured Astrocytes

Cultured astrocytes were plated onto coverslips for electrophysiological experiments. The standard solution for the pipette contained (in mM): 150 KCl, 1 CaCl_2_, 1 MgCl_2_, 5 EGTA, and 10 HEPES (pH 7.2, adjusted with KOH). Standard bath solution contained (in mM): 150 NaCl, 3 KCl, 2 CaCl_2_, 1 MgCl_2_, 10 HEPES, 5.5 D-glucose, and 20 sucrose (pH 7.4, adjusted with NaOH). Patch pipettes were made from borosilicate glass capillaries (Warner Instruments, Washington, DC, USA). The pipette resistance was 5–6 MΩ. Whole-cell currents were recorded using a patch clamp amplifier (Axopatch 700B, Axon Instruments, Union City, CA, USA). Current–voltage relations were measured by applying ramped pulses (from −150 mV to +50 mV over 1000-ms) from a holding potential of −60 mV. A Digidata 1550 A interface (Axon Instruments, Union City, CA, USA) was used to convert digital–analog signals between the amplifier and computer. Data were sampled at 5 kHz and filtered at 1 kHz. Currents were analyzed with Clampfit software (Axon Instruments, Union City, CA, USA). The chamber (RC-25 chamber, Warner Instruments, Washington, DC, USA), which had a volume of 1 mL, was continuously perfused with bathing solution at a rate of 1 mL/min. Different concentrations of spadin were sequentially bath-applied from low concentration to high concentration by switching solenoid valves using a gravity-fed perfusion system (VC-6M, Harvard Apparatus, Holliston, MA, USA). Whole-cell currents were recorded after 2 min when spadin-contained bath solutions were exchanged. All experiments were conducted at room temperature of 20–22 °C.

### 4.9. Electrophysiological Recording in COS-7 Cells

Current–voltage (*I*–*V*) curves were recorded from COS-7 cells expressing TWIK-1 K274E, TREK-1 or concatenated TWIK-1-TREK-1-IRES2-GFP. The standard solution for the pipette contained (in mM): 150 KCl, 1 CaCl_2_, 1 MgCl_2_, 5 EGTA, and 10 HEPES (pH 7.2 was adjusted with KOH). Standard bath solution contained (in mM): 150 NaCl, 3 KCl, 2 CaCl_2_, 1 MgCl_2_, 10 HEPES, 5.5 d-glucose, and 20 sucrose (pH 7.4 was adjusted with NaOH). The *I–V* relations were measured by applying 1-s ramp pulses (from −150 mV to +50 mV) from a holding potential of −60 mV. The Digidata 1550A interface (Axon Instruments, Union City, CA, USA) was used to convert digital–analogue signals between amplifier and computer. Data were sampled at 5 kHz and filtered at 1 kHz. Currents were analyzed with Clampfit software (Axon instruments, Union City, CA, USA). All experiments were conducted at room temperature of 20–22 °C.

### 4.10. Immunohistochemistry

Brain slices were washed with PBS for 20 min at room temperature, followed by antigen retrieval with 10 mM sodium citrate buffer at 85 °C for 30 min. The slices were washed with PBS followed by permeabilization with 0.4% Triton X-100 in PBS at RT for 20 min. Then, the slices were blocked with 10% donkey serum and 0.1% Triton X-100 in PBS at RT for 3 h followed by incubation with primary antibodies, 5% donkey serum, and 0.1% Triton X-100 in PBS at 4 °C overnight. After washing with 0.1% Triton X-100 in PBS at RT for 15 min three times, secondary antibodies, 5% donkey serum, and 0.1% Triton X-100 in PBS at 4 °C were added for 2 h. The slices were counterstained with DAPI and mounted with mounting media (Vectashield, Vector Laboratories Inc., Burlingame, CA, USA). The following antibodies were used: rabbit anti-TREK-1 (Alomone Labs, Jerusalem, Israel, 1:200; RRID: APC-047, 1:200), rat anti-GFAP (ThermoFisher, Waltham, MA, USA; 1:500, RRID: 13-0300, 1:500), and Alexa Fluor 488- and 594-conjugated secondary antibodies (Jackson ImmunoResearch, West Grove, PA, USA; 1:300). All images were acquired using a Nikon Ti2 confocal microscope (Nikon Instruments Inc., Melville, NY, USA).

### 4.11. Lentivirus Construction and Electrophysiological Recording in Hippocampal Slices

Using lentiviral pSicoR vectors expressing validated mouse shRNAs against *TWIK-1* and *TREK- 1*, high-titer lentiviruses were produced by KIST virus facility (Korea) as previously described [11]. Lentiviruses were injected into hippocampal CA1 region (AP: 0.17, ML: 0.17, DV: 0.19) of 7–8 week-old wild-type C57BL/6 mice. The virus-infected mouse brain slices (350 μm) containing the hippocampus were CaCl2, 26 NaHCO3, and 10.0 D-glucose. Individual hippocampal slices were transferred to a recording chamber, which was constantly perfused with artificial cerebrospinal fluid (ACSF) recording solution containing (in mM) 130 NaCl, 24 NaHCO_3_, 3.5 KCl, 1.25 NaH_2_PO_4_, 1.5 CaCl_2_, 1.5 MgCl_2_, and 10 glucose saturated with 95% O_2_–5% CO_2_ at pH 7.4. Brain slices were co-loaded with SR-101 (Sigma Aldrich, St. Louis, MO, USA; 1 mM) dye to identify the location of astrocytes in the CA1 region. The slices were recovered at room temperature for at least 1 h before SR101 incubation and electrophysiological recording. Patch pipettes had a resistance of 5–7 MΩ when filled with pipette solution containing (in mM) 140 KCl, 10 HEPES, 5 EGTA, 2 Mg-ATP, and 0.2 Na-GTP, adjusted to pH 7.4 with KOH. Whole-cell patch recordings were performed on hippocampal astrocytes with a voltage-clamp configuration using an Axopatch 700B (Axon Instruments, Union City, CA, USA).

### 4.12. Statistics

All data are presented as means ± standard error of the mean (s.e.m). The significance of data for comparison was assessed by Student’s *t*-test (paired *t*-test) or one-way ANOVA followed by Turkey’s post hoc test, and significance levels are given as: n.s: not significant, * p < 0.05, ** p < 0.01, *** p < 0.001, and **** p < 0.0001. To obtain the IC_50_ value for dose-dependent inhibition, concentration–response data were fitted with the Hill equation. Prism9.0 software (GraphPad Software, San Diego, CA, USA) was used for carrying out the statistical analysis.

## Supplementary Materials

Supplementary Materials can be found at https://www.mdpi.com/1422-0067/21/24/9639/s1. Figure S1: Spadin does not alter K^+^ conductance in astrocytes knocked down TWIK-1 or both of TREK-1 and TWIK-1.

## Abbreviations

RMP	Reversal membrane potential
4-AP	4-aminopyrimidine
TEA	Tetraethylammonium
shRNA	Short hairpin-forming interference RNA
CRISPR/Cas9	Clustered regularly interspaced short palindromic repeats/CRISP-associated endonuclease 9
sgRNA	Single guide RNA
AAV	Adeno-associated virus
AA	Arachidonic acid
K2P	Two-pore domain K^+^ channel
KO	Knockout
KD	Knockdown
TKDC	*N*-(4-cholorphenyl)-*N*-(2-(3,4-dihydrosioquinolin-2(1H)-yl)-2-oxoethyl)methanesulfonamide
ACSF	Artificial cerebrospinal fluid
