# Supplementary material for: Spadin Modulates Astrocytic Passive Conductance via Inhibition of TWIK-1/TREK-1 Heterodimeric Channels
Source: Int J Mol Sci. 2020 Dec 17;21(24):9639. doi: 10.3390/ijms21249639 (PMC7765948; doi:10.3390/ijms21249639)
Supplement: Supplementary file 1 [file ijms-21-09639-s001.pdf]

International Journal of
Molecular Sciences
MDPI

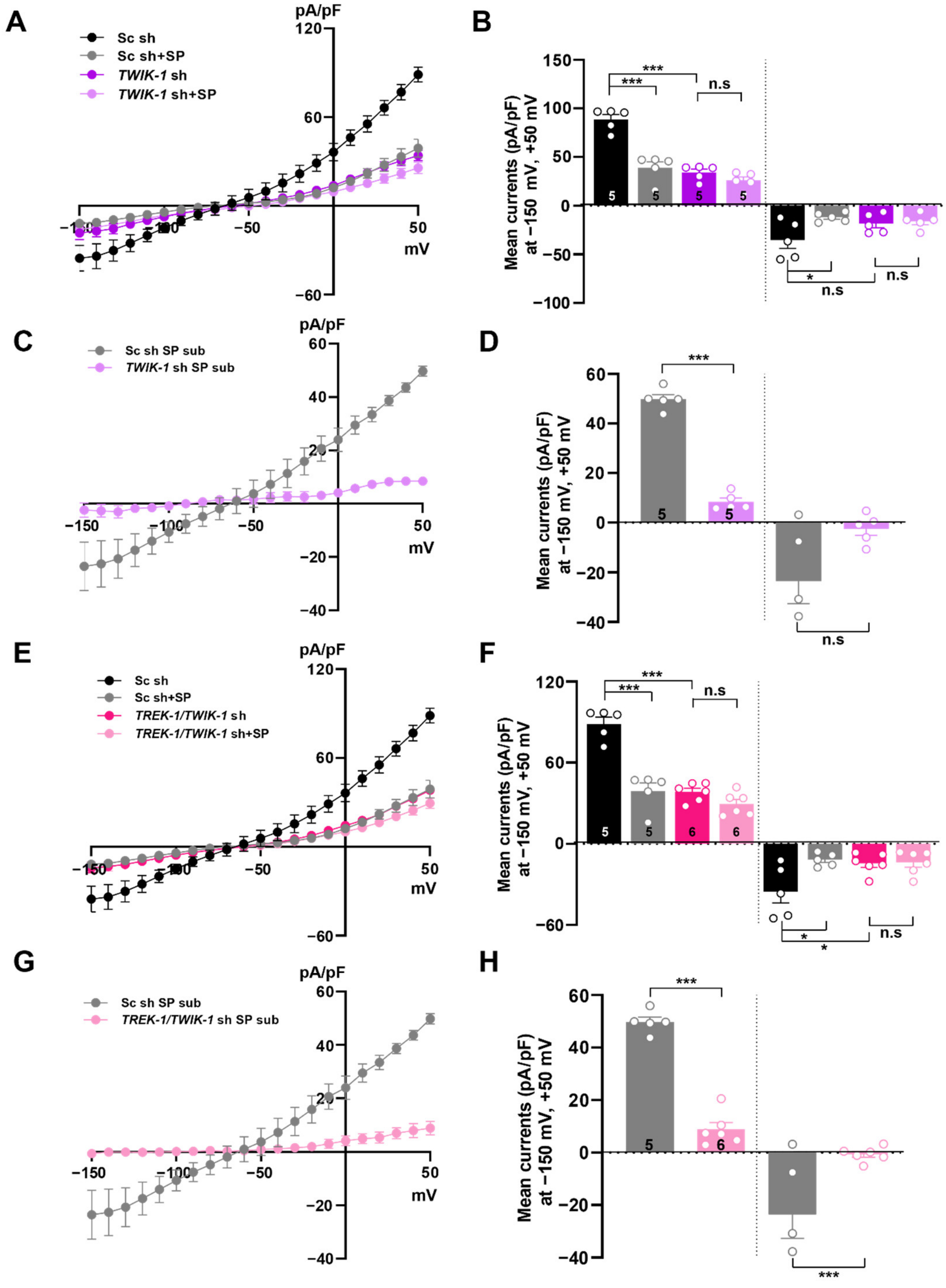

Figure S1. Spadin does not alter K+ conductance in *TWIK-1* or both *TREK-1* and *TWIK-1* knock-down astrocytes. (A) K+ current densities were recorded in primary astrocytes transfected with scrambled shRNA (sc sh) or *TWIK-1* shRNA before and after 10 μM spadin (SP) treatment. (B) Summary histogram showing averaged current densities in each group in (A) at -150 mV and +50 mV. Number on each bar indicates *n* for each condition. All values are mean ± s.e.m. *P*-values were obtained with one-way ANOVA followed by Turkey's post hoc test. n.s: not significant, *$P < 0.05$ and ***$P < 0.01$. (C) Spadin-sensitive currents were calculated by differences between currents before and after spadin treatment in (A). (D) Summary histogram indicating averaged spadin-sensitive currents from the plot in (C) at +50 mV and -150 mV. All values are mean ± s.e.m. *P*-values were obtained with Student's t-test. n.s: not significant, ***$P < 0.001$. (E) Current densities were measured in astrocytes transfected with scrambled control shRNA (Sc shRNA) or *TREK-1* shRNA/*TWIK-1* shRNA before and after

spadin administration. (F ~ H) Summary plot and bar graphs showing the same parameters as in (B ~ D), respectively. Number on each bar indicates *n* for each condition. All values are mean ± s.e.m. *P*-values were obtained with one-way ANOVA followed by Turkey's post hoc test or Student's t-test. n.s: not significant, **P* < 0.05 and ****P* < 0.001.

© 2020 by the author. Licensee MDPI, Basel, Switzerland. This article is an open access article distributed under the terms and conditions of the Creative Commons Attribution (CC BY) license (http://creativecommons.org/licenses/by/4.0/).
